# Piloting a Clinical Decision Support System for Unintended Weight Loss in Primary Care: Mixed Methods Study on Early Cancer Detection

**DOI:** 10.2196/90885

**Published:** 2026-07-28

**Authors:** Javiera Martinez-Gutierrez, Sophie Chima, Barbara Hunter, Alex Lee, Lucas De Mendonca, Deborah Daly, George Fishman, Fong Seng Lim, Benny Wang, Craig Nelson, Brian Nicholson, Jo-Anne Manski-Nankervis, Jon Emery

**Affiliations:** 1 Department of General Practice and Primary Care, Melbourne Medical School, Faculty of Medicine, Dentistry, and Health Sciences, The University of Melbourne, 3rd Floor Medical Building, Cnr Grattam Street and Royal Parade, Melbourne, Victoria, 3010, Australia, 61 136352; 2 Collaborative Centre for Genomic Cancer Medicine, The University of Melbourne, Melbourne, Victoria, Australia; 3 Department of Family Medicine, Faculty of Medicine, Pontificia Universidad Catolica de Chile, Santiago, Chile; 4 Primary Care Collaborative Clinical Trials Group, Melbourne, Victoria, Australia; 5 National University Polyclinic, Singapore, Singapore; 6 Singapore Primary Care Cancer Network (SPriNT), Singapore, Singapore; 7 Western Health Chronic Disease Alliance, Western Health Melbourne, Melbourne, Australia; 8 Department of Medicine – Western Health, The University of Melbourne, Melbourne, Victoria, Australia; 9 Department of Primary Care Health Sciences, University of Oxford, 10. Nuffield, Oxford, England, United Kingdom; 10 Primary Care and Family Medicine Department, Lee Kong Chian School of Medicine, Nanyang Technological University, Singapore, Chile; 11 NHG Polyclinics, National Healthcare Group, Singapore, Singapore

**Keywords:** digital health, primary care, cancer early detection, general practice, clinical decision support

## Abstract

**Background:**

Delayed cancer diagnosis leads to poorer outcomes. Unintended weight loss (UWL) is a nonspecific symptom associated with cancer and other serious conditions, which can make it complex to identify the underlying cause. Clinical decision support systems (CDSSs) can provide evidence-based recommendations to facilitate timely investigation and diagnosis.

**Objective:**

This study aimed to pilot the implementation of a CDSS for improving early cancer detection in primary care patients with UWL.

**Methods:**

Five practices reviewed patients identified by the UWL CDSS. Staff were interviewed on the acceptability and feasibility of the CDSS. Interviews were analyzed thematically using 2 relevant frameworks: the acceptability of health care interventions and the sociotechnical model for evaluation of digital interventions framework. Clinical audits assessed the correct identification of UWL, follow-up rates, and patient characteristics.

**Results:**

Of 60 patients identified by the CDSS as potentially having UWL, 36 (60%) had true UWL. Among the misclassified cases, most patients (16/55, 30%) were intentionally trying to lose weight; this intention was documented as free text in the clinical notes for 98% (58/60) of patients and in a structured field for only 20% of patients. Of the 36 patients, 5 (14%) were actively recalled by their practices for further follow-up; the others (31/36, 86%) were not recalled, as most were deemed already under appropriate follow-up. By 6 months, 94% (34/36) of the cohort had received follow-up care regardless of whether they had been formally recalled. One-third (12/36, 33%) of patients had no additional symptoms, while 36% (13/36) had recorded additional abdominal symptoms. Mental health conditions accounted for 19% of diagnoses linked to UWL consultation. Practice staff were generally receptive to the UWL CDSS concept. It was particularly appreciated by practices with strong quality improvement processes and prior CDSS experience. A high proportion of misclassified patients, poor workflow integration, and conflicting patients’ agendas were cited as implementation barriers.

**Conclusions:**

Our study revealed challenges and potential benefits of implementing a CDSS for identifying patients with UWL at risk of cancer in primary care. While integration issues and misclassification of patients were noted, high rates of follow-up care were observed regardless of the CDSS. These high follow-up rates prompt consideration of whether they reflect Australian primary care and whether a CDSS with these characteristics is the most appropriate approach to support early cancer detection. Future research should focus on improving CDSS integration with existing workflows, enhancing correct identification through access to clinical notes and the use of more sophisticated digital methods (eg, AI). These findings highlight the potential of CDSS in improving patient care and the complexities involved in their successful implementation.

## Introduction

Early detection of cancer is crucial for improving patient outcomes, offering several significant benefits. It can substantially enhance a patient’s chance of survival, broaden the range of available treatment options, and contribute to an improved quality of life [1,2]. Moreover, timely diagnosis can help reduce the overall burden on health care systems by potentially decreasing the complexity and cost of treatments required for advanced-stage cancers [3]. General practice plays a pivotal role in this early detection process, often serving as the first point of contact for patients experiencing new signs or symptoms of cancer [4,5]. However, the diagnosis of cancer can be particularly challenging in many cases, given the nonspecific nature of presenting symptoms. These symptoms can often be associated with a number of other, more common and less severe conditions, making it difficult for general practitioners (GPs) to immediately identify potential malignancies [6].

Unintended weight loss (UWL) is an example of a nonspecific symptom associated with cancer. While UWL can also be associated with various benign conditions, including depression, thyroid disorders, and gastrointestinal diseases, it is also a potential indicator of several cancer types [6,7]. The diagnostic complexity is compounded by the fact that UWL can initially occur in isolation, without accompanying clear symptoms that might point toward a specific underlying cause [8]. This absence of additional clinical indicators makes it particularly challenging for health care professionals to determine the optimal route of investigation. The nonspecific nature of UWL as a symptom underscores the need for a comprehensive and systematic approach to patient evaluation, potentially incorporating advanced diagnostic tools and risk assessment models.

Given the diagnostic complexity of nonspecific symptoms’ association with many cancers, clinical decision support systems (CDSSs) are being developed for use in general practice [9]. These tools are designed to assist GPs in making more informed decisions by integrating algorithms underpinned by patient data and clinical guidelines [10]. In the context of early cancer detection, CDSS can potentially streamline the diagnostic process by identifying patients who require further investigation or referral [11]. For patients presenting with nonspecific symptoms such as UWL, these tools could be particularly valuable in stratifying risk and supporting decision-making.

Studies evaluating CDSS in practice have demonstrated increased adherence to guidelines and successful diagnostic support [11]. However, the majority of developed tools fail to achieve widespread implementation, and those that are implemented often face low uptake rates [12]. While there are significant opportunities to support early cancer detection, designing tools that are useful, acceptable, and seamlessly integrated into primary care workflows is a complex, multistep process. This process necessitates continuous input and refinement from end users throughout the development cycle to ensure relevance and usability [13,14]. This study aimed to evaluate a novel CDSS designed for the early detection of cancer in patients presenting to general practice with UWL. Specifically, the study sought to assess whether the tool was acceptable to practitioners, met the diverse needs of general practice, and effectively supported the delivery of high-quality, evidence-based care.

## Methods

### Design

This study used mixed methods to evaluate the implementation of a complex CDSS for patients with UWL, using patient data collected in clinics through interviews with participating general practices and an electronic health record (EHR) audit.

### The Future Health Today Tool

Future Health Today (FHT; The University of Melbourne) is a software platform that has been developed for use in general practice, streamlining the identification and management of chronic diseases. While the development of the tool has been described elsewhere, it underwent an extensive co-design process with GPs and consumers [15,16]. FHT is integrated with the EHR and has 2 core components: First, it is a CDSS that provides proactive, guideline-based recommendations at the point of care; the second component is a web-based portal, which supports quality improvement (QI) activities in general practice, has the ability to audit and recall patient groups within the practice population, and provides access to education and resources.

### The UWL Module

The FHT tool supports a wide range of conditions, and different modules are developed for use in FHT [17,18]. This study explores the use of the FHT UWL module, which flags patient records with UWL indicated in the EHR in the past 6 months. Development of the UWL module was extensive, beginning with the development and validation of phenotyping and risk prediction algorithms to identify patients with “unintended weight loss” terms in structured EHR fields (reason for visit, diagnosis, and prescriptions) while also excluding those with indications of intentional weight loss. Additional exclusion criteria, including recent cancer diagnoses, intentional weight loss reasons (eg, bariatric surgery or weight loss prescriptions), and/or increasing weight measurements, were further added [19]. We did not use actual weight loss as an inclusion criterion, given the low prevalence of weight measurements in Australian general practice data [20]. Algorithm criteria (age, sex, blood test results, and smoking status) determined the patient’s risk profile and provided the recommendation delivered to the GP.

The phenotyping and validation process and the risk profile calculations have been described elsewhere [19]. Briefly, after applying inclusion and exclusion criteria to identify patients with UWL, the module provided 3 types of recommendations according to cancer risk:

1. For patients with low cancer risk (<2%): a recommendation to take new weight measurements and a reminder of the most common differential diagnosis according to age and sex
2. For patients with moderate to high risk (>2%) and absent or normal blood test results: recommendation to ask for symptoms and order (additional) pathology tests
3. For patients with moderate to high risk (>2%) and abnormal blood test results: recommendations to follow up with further testing (imaging, scopes, and referrals)

**Figure 1.**
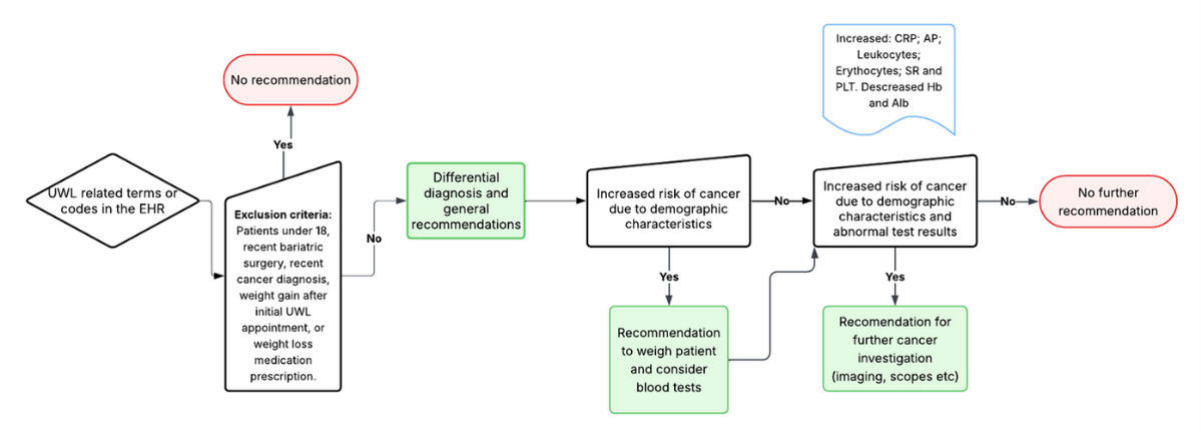
Unintended weight loss (UWL) module algorithm rules and recommendations. Black boxes represent the algorithm processes; green boxes are relevant recommendations; blue box provides further relevant extra information; red boxes represent no further recommendations. Alb: albumin; AP: alkaline phosphatase; CRP: C-reactive protein; Hb: hemoglobin; PLT: platelets; SR: sedimentation rate.

Figure 1 shows a simplified diagrammatic representation of the rule-based algorithm rules.

**Figure 2.**
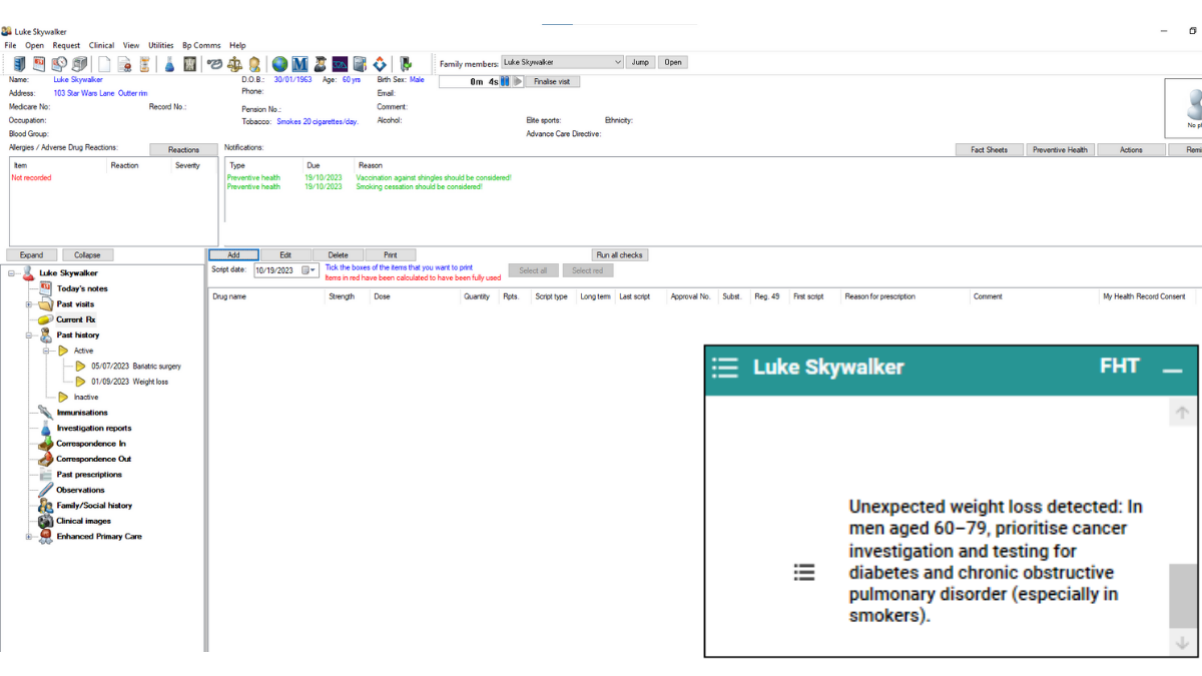
Example of a recommendation as it appears in a medical record (hypothetical patient).

The content and wording of the recommendations and the supporting resources were developed with input from GP and consumer and were informed by evidence from a previous scoping review [21]. An example of a recommendation as it appears in the EHR is shown in Figure 2.

### Study Participants, Recruitment, and Implementation

Recruitment of general practices occurred by approaching practices participating in the FHT program (ie, practices that already had FHT installed). Practice managers and/or practice owners were contacted and provided with study information. All recruitment documentation was created with assistance from the FHT Consumer and General Practice Advisory Group. In agreeing to participate in the study, practices were required to review a list of patients with UWL, nominate 1 person from the practice to participate in an interview, and partake in a clinical audit.

We invited, via email, practices that had at least five patients identified by the UWL algorithm. Practices recruited to participate in the pilot UWL implementation study had the FHT UWL module switched on for at least 6 months. At study initiation, the FHT team created a list of patients (also referred to as "cohort") who met the criteria for UWL, which could be accessed via the FHT web-based portal. Practices were also offered a 30-minute, Zoom (Zoom Communications, Inc)–based training session once the module was switched on. Each practice nominated an appropriate person (eg, GP, general practice nurse, and QI lead) or team of people to review the cohort of patients with UWL. The purpose of the cohort review was to determine which patients should be recalled for further follow-up or whether the recommendations should be deferred. After review of the patient list, practices were asked to fill in a log with the kind of action performed (eg, patient recalled or deferred) and the reasons for this action (eg, weight loss was intentional, patient is already being followed up, and patient requires further investigation). After the cohort review was complete, practices were invited to use FHT as they chose for the remainder of the study.

### Data Collection

#### Interviews

One month after the implementation of the UWL module and completion of the cohort review, practice staff who reviewed the cohort of patients flagged with UWL were contacted to participate in an interview. At least one person from each general practice participated in a semistructured interview conducted via Zoom. Interview schedules (Multimedia Appendix 1) were informed by relevant literature and prior qualitative work conducted as part of the FHT program [22,23]. In the interview, staff were asked about the FHT technology generally, their experience using the UWL module to review and recall patients, and the UWL recommendations. The interviews explored the themes of implementation (including barriers and facilitators to use), the content and clinical appropriateness of the UWL recommendations, and usability, acceptability, and feasibility of the module in the longer term. All interviews were audio recorded for analysis and were transcribed using Otter.AI. JMG conducted the interviews. Interview duration was 30 to 45 minutes.

JMG is a female mid-career researcher and academic GP with experience in qualitative research. SC is an early-career researcher with expertise in FHT and qualitative research. JMG did not have a previous relationship with any of the interviewees. SC had previous contact with 2 practice staff from 1 practice as a research coordinator for a previous study using FHT. We did not provide information regarding personal goals or additional reasons for doing the research other than the description in the plain language statement. Participants were not asked to review interview transcripts or study findings, and no repeat interviews were conducted. Field notes were not recorded during or after the interviews. To ensure methodological rigor and transparency, the presentation of findings adhered to the COREQ (Consolidated Criteria for Reporting Qualitative Research) checklist (Checklist 1).

#### Clinical Audits

An EHR audit was conducted approximately 1 to 4 months after the creation and review of the UWL cohort. EHR data of patients who were flagged by the FHT platform as having UWL were audited by a clinical researcher using a data extraction template. The extraction template was piloted and refined by clinician researchers in the team before data collection (Multimedia Appendix 2). Data extracted from the EHR included age, sex, details on the record of UWL and all UWL-related free text, prescriptions, laboratory results, referrals, images, weight measurements, and any UWL-related follow-up and were entered into the REDCap platform [24]. The time frame of extracted data included the 6 months prior to the initial UWL presentation until the date of the clinical audit. Due to the remoteness or location of participating practices, clinical audits were conducted using TeamViewer, a secure remote access software hosted by the University of Melbourne.

### Data Analysis

#### Interviews

**Figure 3.**
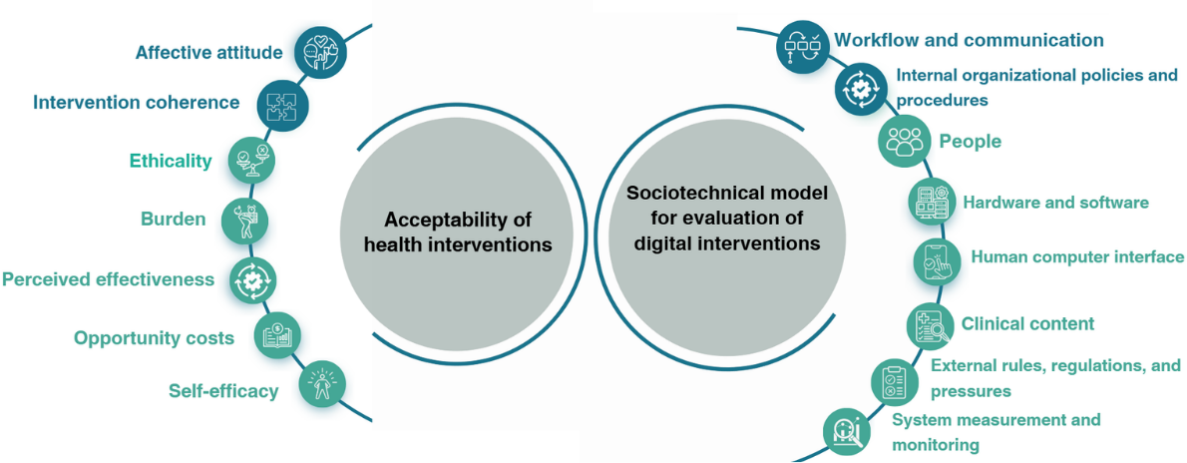
Frameworks used in the analysis of semistructured interviews with general practice.

Interview transcripts were uploaded into NVivo 14 (Lumivero) and thematically analyzed independently by 2 researchers (JMG and SC) [25]. We used deductive thematic analysis using 2 overarching frameworks in the analysis: acceptability of health care interventions [26] and sociotechnical model for evaluation of digital interventions framework [22] (Figure 3).

We chose these 2 frameworks for their health care–specific focus and comprehensive scope, encompassing both technical and human factors in CDSS implementation. These recent frameworks offer a multidimensional approach to assess user perceptions and evaluate digital interventions, allowing us to identify a broad range of barriers and facilitators for safe CDSS testing in primary care. In addition, inductive analysis was conducted to create de novo themes developed from the data. The qualitative themes mapped onto the 2 frameworks are reported based on dominant themes arising from the interviews. Differences in interpretation were discussed and resolved by consensus. Input was sought from the wider research team, including consumer investigators, in the development and interpretation of themes.

Main themes analyzed are shown in blue, which are adapted from studies by Sekhon et al [26] and Sittig and Singh [22].

#### Clinical Audits and Implementation Data

Data collected in the EHR audit were uploaded to REDCap and then downloaded into a master Excel (Microsoft) spreadsheet to be reviewed, cleaned, and analyzed by 2 researchers (JMG and SC), including 1 clinician (JMG). Disagreements were discussed with the wider clinical and implementation team. Data collected via the EHR audit and clinical review log for each practice were analyzed using descriptive statistics. This included determining the binary outcomes: “Does this patient have unexpected weight loss?” (yes/no) and “Has this patient been followed up for UWL?” (yes/no). True and false positives of UWL were calculated using the information collected in the EHR audit following a predetermined set of criteria (Multimedia Appendix 3). For patients with true UWL, descriptive statistics were used to describe follow-up variables in the coded and free-text fields (diagnosis, symptoms, investigations, and referrals).

### Ethical Considerations

Ethical approval was granted by the University of Melbourne Human Research Ethics Committee STEMM 1 (Ethics ID: 25308). Participants were provided with a plain language statement explaining the study. Informed consent was obtained from all participating general practices. Practices were paid Aus $1000 (approximately US $660) for participation. Participation was voluntary, and all interview participants were reimbursed for their time (Aus $100, equivalent to US $67).

## Results

### Practice Characteristics

We recruited 5 general practices from a pool of 40 practices that had the FHT program installed. Practices were located in Queensland and Victoria, Australia. Four practices were located in metropolitan areas, and 1 practice was located in a rural area based on the Australian Modified Monash Model [27]. The mean number of patients identified as having UWL 6 months previous to cohort creation was 13 (SD 5) patients per practice. Practice characteristics are described in Table 1.

**Table 1.** Characteristics of 5 general practices participating in pilot study (n=5).

Practice characteristics	Values
Number of active patients^a^ in each practice, mean (SD)	17,619 (3368)
Number of active patients by reported gender, mean (SD)	
Male	7623 (1325)
Female	9505 (1891)
Other	491 (310)
Number of active patients aged ≥40 y, mean (SD)	8933 (1905)
Number of physicians per practice, mean (SD)	10.4 (5)
Rurality classification of practice^b^, n (%)	
Metropolitan	4 (80)
Rural	1 (20)
State or location, n (%)	
Victoria	2 (40)
Queensland	3 (60)
Cohort size, mean (SD)	13 (5)

a The Royal Australian College of General Practitioners defines an “active patient” as “a patient who has attended the practice 3 or more times in the past 2 years” [28].

b Rurality classification based on the Modified Monash Model [27].

### Practice Staff Interviews

We conducted 5 interviews with 7 practice staff. Interviews were conducted via Zoom after the list of patients had been reviewed by practice staff and at least a month had passed to give patients the opportunity to come back for a follow-up appointment if they had been recalled by the practice.

Participant characteristics are presented in Table 2.

**Table 2.** Characteristics of interview participants.

Characteristics	Values, n (%)
Age	
20‐29	1 (14)
40‐49	3 (43)
50‐59	1 (14)
≥60	1 (14)
Missing	1 (14)
Gender	
Male	1 (14)
Female	6 (86)
Role	
GP^a^	1 (14)
PN^b^	3 (43)
Pharmacist^c^	1 (14)
PM^d^ or practice owner	1 (14)
Admin^c^	1 (14)
Practice setting	
Metro	5 (71)
Rural	2 (29)

a GP: general practitioner.

b PN: practice nurse.

c Role also involves leading the quality improvement activities in the practice.

d PM: practice manager.

We first describe “affective attitude” to contextualize the staff’s general attitude toward the CDSS. Furthermore, 3 main deductive themes became evident from our analysis using the 2 frameworks: internal organizational policies, procedures, and culture; and workflow and communication and intervention coherence. Remaining relevant themes from the frameworks are summarized, with exemplary quotations provided in Multimedia Appendix 4.

### Affective Attitude

Overall, participants liked the concept of the tool. For quality assurance purposes, the cohort tool, with the ability to create lists of patients with specific conditions and the detailed information it provided on the patient, was found to be very useful. Regarding the UWL cohort, they particularly liked that the cohorts were composed of only a few patients, as expected given the low prevalence (1.5%) of UWL reported in the literature.

Clinical and administrative staff generally favored QI initiatives and supporting software, with 1 staff member reporting frequent use of FHT in management plans and health assessments.

One clinical staff member mentioned finding both features useful:

*I like the way that it pops up a reminder in real time when we open their record. And it’s really useful to be able to develop the cohorts for auditing purposes and for purposes of developing, you know, PDSA (Plan, Do, Study, Act) cycle quality improvement activities and things like that. So, so overall, I’ve found FHT really, a really useful innovation.*

[Clinical staff, practice 2]

*I like when you click on the patient’s name in the cohort, you know you can see the meds, there’s a snapshot of their history. I like the MBS (Medicare Benefits Schedule) items that might apply, because part of my job is making sure we’re sustainable and maximizing those, those chronic disease item opportunities.*

[Clinical staff, practice 1]

### Intervention Coherence

Despite FHT being installed for at least a year in all 5 practices, 5 (70%) participants were not well acquainted with the cohort tool in FHT. Even after co-design efforts, provision of written materials, and virtual training offers, several challenges emerged in the implementation of the UWL module. The inclusion and exclusion criteria of the algorithm were not well understood. Users were uncertain about how patients progressed through the patient management system (from “awaiting action” to “deferred,” “actioned,” or “no further action needed”), indicating confusion about this component of FHT.

After reviewing the lists, clinicians identified that several patients on the list were intentionally trying to lose weight (see “Clinical Audits” section) or had only one recorded weight measurement and staff were confused by the inclusion of these patients. Technical issues further complicated matters, as the tool malfunctioned during use by the first practice. In this case, the practice staff reviewed the cohort and actioned the patients accordingly (marking them as “deferred”: no action needed or “recalled”), but the system did not save these actions, causing all patients to remain in the “awaiting action” category, causing additional confusion.

Despite these challenges, there were positive aspects. The tool’s visual elements and patient summaries were seen as helpful facilitators. Most users found reviewing patients in the cohort straightforward:

*I didn’t find it too hard. It was - once you went into the patient’s file it was pretty self-explanatory if you just kept going through the flow of the appointments and what the doctors - because you can see where it said weight loss in the reason for visit, if you sort of read from then on, and then go back if you have to, but, it sort of flowed pretty well, so you could understand.*

[Clinical staff, practice 3]

*First of all, of the 19 patients, some of them, I’m not sure why they popped up on the list, some of them had only ever had one weight recorded. So I’m not quite sure how the tool came to conclude that they’d lost weight.*

[Clinical staff, practice 2]

### Internal Organizational Policies, Procedures, and Culture

Internal processes arose as the most influential factor in the use of the cohort tool.

GPs typically document most patient information in the free-text clinical notes field of the EHR. However, the CDSS algorithm is currently unable to read these notes, relying only on structured text fields such as “reason for visit” or “diagnosis,” creating a substantial barrier to its effective use. This mismatch between clinician documentation and the CDSS data input requirements limited the tool’s ability to provide comprehensive support.

In 2 practices, the manager asked GPs whether they would be open to a CDSS system that would issue reminder pop-ups at the point of care when a patient presented. They collectively opted not to have it installed as they were concerned about alert fatigue, showing a lack of understanding of how frequently this tool for this algorithm would appear, as UWL is a relatively uncommon presentation.

1. *Dedicated personnel:* Having a dedicated PN or allocated person, such as a pharmacist or practice manager, for QI activities facilitated the use of the cohort tool. In these cases, the integration with the practice workflow was better achieved, ensuring more consistent and effective use of the system. However, designated resources have a cost associated, and many practices do not have sufficient staff available to undertake this type of role.
2. *Existing QI systems:* Some practices already had seemingly effective systems in place for data searches and QI. Two practices, which had the same owner and manager, were very comfortable using the search tool embedded in their own EHR. Although the EHR search tool was simple and would not be able to flag patients with UWL nor identify risk of cancer, the practice manager thought it was good enough for their QI requirements, making the tool seem unnecessary for their needs. They also did not see the use of the point-of-care tool as they had determined it would result in alert fatigue. These established workflows were seen as competing with the new CDSS, depending on how well acquainted the staff were with FHT and whether this UWL algorithm integrated with existing processes.
3. *Varied models of use:* In 3 of the 5 practices, clinical staff conducting audits did not directly engage with the CDSS but instead worked from patient lists provided by practice managers or research coordinators who knew how to use FHT. In these practices, FHT was understood to be mostly a research tool, rather than a tool for everyday QI activities. This indirect use of the system led to a disconnect between the tool’s capabilities and the clinical decision-making process in the clinic. The 2 remaining practices were well acquainted with the tool and were already using it for QI and clinical care activities for other conditions on their own account.
4. *Time allocation:* Time allocation for cohort review varied significantly between roles. Nurses and coordinators had allocated time for QI activities and worked within office hours, while the GP (similar to most GPs in Australia), lacking dedicated time, resorted to after-hours completion, impacting workflow integration.
5. *User familiarity and role integration:* In practices where the same person reviewed the cohort and assessed clinical relevance, there tended to be better integration of the module within the practice workflow. These individuals, often practice managers with clinical roles, were typically more familiar with the tool and could more effectively coordinate follow-up actions with GPs. This familiarity and role integration led to more streamlined use of the CDSS.

Quotes exemplifying each one of these factors or processes can be found in Table 3.

**Table 3.** Internal factors or processes affecting implementation.

Internal factor or process	Selected quotation
Organization culture and staff practices	“No one [GP] uses this drop-down system codes [in the reason for visit field in the EHR] that takes one second” (clinical staff, practice 3). “No, no, I haven’t got [FHT] on for the GPs at all. And the reason for that is most of my doctors are anti pop ups” (practice manager, practice 5).
Dedicated personnel	“So as part of being the nurse coordinator, I do take on a lot of the quality improvement in the practice, so I’m one of the leads on that part” (clinical staff, practice 3).
Existing quality improvement systems	But most of our data searches in that we actually use [our EHR]. So which is, which is the software that we use. So it’s much easier for us to just be working on [our EHR]” (practice manager, practice 4).
Varied models of use	“In the program, so I went, basically made a list of all the patients and all the like possible causes. I gave it to [PM], and then kind of just assessed, like, what was going on with the patient, whether they needed to come back to see the doctor, or most of them were already, like in discussion with the doctor about that weight loss” (practice manager, practice 5).
Time allocation	“I think they just did that out of hours, you know, an extra job to do with an evening, because I sent them, I sent everybody, at least a round of their relevant patients, and asked them to have a look” (clinical staff, practice 2).
User familiarity and role integration	“So the portal, so yep, and like the recalling or deferring patients, so often looking up a lot of if it’s the clinical side, I'll tend to look up all the clinical data and stuff like that, and refer and defer and review patient, bring it to the attention of the doctors if they're coming in for another appointment” (clinical staff, practice 3).

### Workflow and Communication

Workflow and communication factors were described as crucial determinants in the use of the module. This theme, closely intertwined with internal processes mentioned earlier, stood out for both its prevalence and the depth of insights it provided.

The ease of integrating the tool into existing workflows varied among practices. Those with a special interest in data analysis and QI found it easier to embed the cohort tool into their routines. However, the lack of integration of the system with the practices’ message software was a common issue, hindering direct action on strategies such as reminders and recalls.

The process of reviewing the cohort was generally consistent and considered relatively easy across practices.

1. Adaptations and workarounds: Practices developed their own methods for managing the cohort and relaying information, such as using spreadsheets or internal notes. The fact that FHT was adaptable for this was valued and highlights the need for flexible systems that can accommodate various practice styles.
2. Communication challenges: Clinical and administrative staff mentioned that communicating the need for recall to patients could prove challenging, especially when the issue (eg, UWL) was not a priority for patients or when they were unaware of the problem. This indicates that clinician judgment always sits on top of any CDSS and that the provision of a list does not automatically mean it is clinically relevant or preferred to actively recall patients.
3. Training and understanding: Three practices did not fully use all CDSS features (eg, pop-ups) due to lack of experience or training (one practice) or practice decision not to use the pop-up feature to avoid alert fatigue (2 practices). This affected their understanding of the tool’s capabilities and its integration into their workflow.
4. Role-specific challenges: The workflow varied by role, with a GP reviewing pop-ups during consultations but not always acting on them due to existing audit processes. Practice nurses sometimes struggled with determining follow-up needs, highlighting the importance of role-specific training and clear guidelines.

Exemplary quotes:

*[FHT] It doesn’t talk back to [our EHR] to add any reminders or add any actions, and knowing how hard [our EHR] is to integrate with...*

[Clinical staff, practice 1]

*I think it’s useful, but it’s still up to the GPs to sort of use it more? Yeah, I think that’s our, that’s our biggest struggle is to get more of them using it.*

[Clinical staff, practice 3]

*Or if the patient has had no nurse contact, she may not have seen that patient at all. So it would have been a very awkward conversation for her to actually call the patient and go, Hey, how’s it going? Are you trying to lose weight?*

[Administrative staff, practice 4]

### Clinical Audits

The algorithms identified 62 patients with possible UWL. We extracted data from a total of 60 patients. Two patients were excluded from analysis as records could not be accessed, given (1) no longer an active patient and (2) no weight loss recorded as a reason for the visit or in their past history. The tool correctly identified 36 of 60 (60%) patients with UWL established by clinical assessment of the information in the EHR.

Patients with UWL confirmed in the EHR were older and equally distributed by sex, while patients with intentional weight loss recorded in the EHR were younger and mostly women. Intention to lose weight was recorded as free text in the clinical notes in 98% of cases, with only 20% of patients having intention recorded in a structured field (reason for visit or diagnosis). Sixty-one percent (22/36) of patients with UWL were at moderate to high risk of cancer. Five (8%) of 60 patients were recalled for further investigation. Details of patients’ characteristics and CDSS deployment can be found in Table 4.

**Table 4.** Patient characteristics and related clinical decision support system recommendations.

	All patients	Patients with UWL^a^	Patients with intended weight loss
Number of patients, n (%)	60 (100)	36 (60)	24 (40)
Age, median (IQR)	57.5 (35.5)	68.5 (130.5)	44 (21.5)
Sex, n (%)			
Males	27 (45)	18 (50)	9 (37.5)
Females	32 (53)	17 (47)	15 (62.5)
Missing	1 (2)	1 (3)	0 (0)
Recommendations^b^, n (%)			
Patients at low risk of cancer	20 (33)	9 (25)	11 (46)
Patients at moderate and high risk of cancer	30 (50)	22 (61)	8 (33)
No recommendations	10 (17)	5 (14)	5 (21)
Patient recalled for further follow-up, n (%)			
Yes	5 (8)	5 (14)	0 (0)
No^c^	55 (92)	31 (86)	24 (100)
Where was intention to lose weight recorded^d^, n (%)			
Reason for visit	10 (17)	5 (14)	5 (21)
Diagnosis	2 (3)	1 (3)	1 (4)
Clinical notes	59 (98)	35 (97)	24 (100)
Other (inferred from test ordering)	1 (2)	1 (3)	0 (0)

a UWL: unintended weight loss.

b Refer to Figure 1 for definition of risk and recommendations.

c This includes patients for whom the practice determined did not need to be recalled for further follow-up (eg, deferred for review until a later date, already under investigation, known cause of UWL). Reasons for deferral or no action taken are reported in Table 5.

d Intention may have been recorded in more than one electronic health record field.

**Table 5.** Reasons for deferral in 55 patients identified in unintended weight loss (UWL) cohort (n=55).

Deferral reasons	Values, n (%)
UWL with known cause	16 (30)
Incorrectly identified according to practice	
Intentional weight loss	16 (30)
Data error	1 (1.8)
No evidence of weight loss	6 (11)
UWL with unknown cause and already under investigation	7 (12.7)
No reason for deferral given or missing	9 (14.5)

Ninety-two percent (55/60) of all patients were deferred, as practices deemed no further follow-up was needed. Reasons for deferral were divided into three main groups: (1) unintentional weight loss with known cause, (2) patient incorrectly included in cohort (eg, intentional weight loss, data error, and increased weight), and (3) unintentional weight loss with cause unknown being followed up already. Table 5 presents the frequency of reasons for referral.

A summary of clinical characteristics and follow-up of the 36 patients with UWL is described in Table 6 (more details in Multimedia Appendix 5). Following their initial UWL consultation, the vast majority of patients with UWL (34/36, 94%) received comprehensive follow-up care. This care encompassed subsequent GP appointments, specialist referrals, various investigations, and/or imaging studies as needed. On average, patients attended a median follow-up of 8 (IQR 10) visits after their initial UWL consultation until the audit date.

**Table 6.** Summary of clinical characteristics and follow-up of patients with confirmed unintentional weight loss (UWL; n=36 patients).

Follow-up and clinical characteristics	Values
Number of visits after initial UWL visit (6 mo follow-up period), median (IQR)	8 (10)
UWL discussed again after initial UWL visit (6-mo follow-up period), n (%)	
Yes	20 (56)
No	16 (44)
Number of patients followed up after initial UWL presentation?^a^, n (%)	34 (94)
Other presenting symptoms at index date^b^, n (%)	
None	12 (33)
Nonspecific (eg, appetite loss and fatigue)	17 (47)
Gastrointestinal (eg, abdominal pain and vomiting)	13 (36)
Mental health (stress, anxiety, mood, or insomnia)	7 (19)
Respiratory (asthma exacerbation and cough)	3 (8)
Other (eg, oral and urinary symptoms)	8 (22)
Investigations ordered by GP^c^, n (%)	
Yes	31 (86)
No	5 (14)
Investigation ordered^d^, n (%)	
Hematology (full blood count and iron studies)	53 (147)
Metabolic (eg, HbA_1c_^e^ and thyroid function)	90 (250)
Cancer markers (eg, Ca12, CEA, and Ca19-9)	8 (22)
Celiac serology (celiac antibodies)	2 (6)
Vitamins (eg, B12 or folate and vitamin D)	25 (70)
Inflammatory markers (CRP^f^ and ESR^g^)	28 (78)
Other (eg, urine, STIs^h^, and virus)	37(101)
Imaging referral, n (%)	
Yes	22 (61)
No	14 (39)
Imaging test ordered^i^, n (%)	
CT^j^ scan	13 (36)
Colonoscopy	5 (14)
Endoscopy	5 (14)
X-ray	7 (19)
Ultrasound	3 (8)
Other	2 (6)
Referrals, n (%)	
Yes	20 (56)
No	16 (44)
Referrals (detail)^k^, n (%)	
General surgeon for colonoscopy	4 (11)
General surgeon for gastroscopy	3 (8)
Gastroenterology for endoscopy	2 (6)
Other	16 (44)
Related diagnoses within 6 months of UWL appointment to audit date recorded^l^, n (%)	
Yes	20 (56)
No	16 (44)
Comorbidities^m^, n (%)	
Mental health conditions	7 (19)
Cancer	4 (11)
Chronic obstructive pulmonary disease	3 (8)
Other	9 (25)

a Includes another consultation where UWL was discussed and/or any investigations, imaging, or referral after the index date.

b Patients may have had more than one symptom recorded at or after index date.

c GP: general practitioner.

d Patients may have had more than 1 investigation ordered.

e HbA_1c_: hemoglobin A_1c_.

f CRP: C-reactive protein.

g ESR: erythrocyte sedimentation rate.

h STI: sexually transmitted infection.

i Some patients had more than one imaging request.

j CT: computed tomography.

k Patients may have been referred to more than 1 specialty.

l “Related diagnosis” defined by the practice GP in the electronic health record as a possible reason for UWL recorded within 6 mo of UWL (new or preexisting).

m Patients may have had more than 1 diagnosis recorded.

One-third of patients (12/36, 33%) presented to the first UWL visit with UWL as their only symptom. Almost half of patients (17/36, 47%) presented with other nonspecific symptoms such as appetite loss and lethargy, and the most common accompanying specific symptoms were gastrointestinal symptoms (13/36, 36%), followed by mental health symptoms (7/36, 19%).

Eighty-six percentage (n=31) of patients had been investigated with blood tests. The most common tests ordered were full blood count tests (n=30, 83%), liver function tests (n=30, 83%), creatinine testing (n=25, 69%), iron studies (n=23, 64%), calcium testing (n=22, 61%), and thyroid function tests (n=18, 50%).

Diagnostic imaging and endoscopic procedures were frequently used in the investigation of UWL. Nearly two-thirds (22/36, 61%) of patients underwent imaging studies or endoscopic examinations. Of these, more than half (13/22, 59%) underwent CT imaging, which included abdominal, lung, and/or pelvic regions.

While direct colonoscopy referrals were relatively low (5 patients), a more common approach was to refer patients to specialists for consideration of endoscopic procedures. One-quarter (9/36, 25%) of patients were referred to either a gastroenterologist or, in rural practices, a general surgeon for consideration of upper and/or lower endoscopy.

More than half of the patients (20/36, 56%) received a diagnosis related to their UWL, as determined by their GP and documented in the EHR. The most prevalent diagnoses associated with UWL were mental health conditions, followed by cancer.

Four (11%) of 36 patients were diagnosed with cancer, including chronic lymphocytic leukemia, myeloma, breast cancer, and renal cancer. However, only the renal cancer case was diagnosed following the UWL consultation. The other 3 (8%) patients should have been excluded from the study based on the predefined algorithm, as these diagnoses were made prior to the UWL visit and within the preceding 5 years.

## Discussion

### Principal Findings

Our study aimed to pilot the implementation of a CDSS to identify patients with UWL at risk of cancer in primary care settings. This initiative involved extensive preliminary work, including the use of primary care databases to identify patients with UWL and calculate their cancer risk using a UK-developed risk prediction model [19]. We also co-designed recommendations and resources and tested the CDSS in simulated scenarios before implementation. The initial findings suggested that the CDSS would be both acceptable and feasible, prompting us to proceed with the implementation (unpublished data).

The implementation process involved asking clinical staff to review a list of patients flagged by the tool and make decisions to defer or recall patients based on their clinical expertise. Using 2 relevant frameworks [22,26], our analysis of staff interviews revealed that most found the concept of identifying patients with UWL at risk of cancer acceptable, and the review process was easy to understand. The most influential factors affecting specific use were the practices’ internal processes, culture, workflow, and communication practices.

We observed 2 distinct opinions regarding the use and feasibility of the CDSS for implementation. Although FHT had been installed and used by all practices for over a year, staff familiar with QI activities within the FHT program found the tool useful and feasible. They reported that the process was not burdensome and could be integrated into their usual workflow. Conversely, practices unfamiliar with the tool found it less helpful, citing high misclassification of patients with intentional weight loss, lack of integration with their EHR, and inability to perform recalls through their regular workflow. This dichotomy aligns with existing literature, which identifies trust in the tool and seamless integration into workflow as major factors influencing CDSS implementation success [11]. To overcome some of these barriers, a module such as this could be made actionable within the EHR (recalls, tasking, and documentation editable directly from the cohort view) and provide configurable, role-specific triggers to reduce irrelevant pop-ups (which are a known cause of alert fatigue) [29]. Operational measures include role-tailored training, clear follow-up protocols, and decision aids for practice nurses [30]. At the organizational level, provenance and governance processes, local champions, piloted roll-outs across diverse practice types, and explicit evaluation of implementation outcomes (adoption, fidelity, and acceptability) using implementation frameworks (eg, Normalization Process Theory and Proctor’s outcomes) will improve representativeness and scalability [31,32]. Together, these levers—designed and evaluated in partnership with frontline staff—should make integration more seamless, reduce cognitive burden, and increase the likelihood that the tool supports safe, equitable care across a wider range of practices.

Another significant barrier was the lack of allocated time for QI activities, particularly for GPs. This finding is consistent with broader literature on the challenges of implementing QI initiatives in primary care settings [33,34].

Clinical EHR audits showed impressively high follow-up care rates, with 94% (34/36) of patients with confirmed UWL receiving appropriate follow-up and a median of 8 follow-up visits per patient. Our study revealed an exceptionally high follow-up rate, with over 50% (20/36) of referrals made to various specialists. Australian and international literature reports delays in referral for unspecific symptoms compared with specific symptoms [35,36]. However, our findings are consistent with results from our previous randomized controlled trial that used FHT to identify patients with abnormal test results at risk of cancer. In that study, even control practices demonstrated follow-up rates exceeding 70% [17].

The high follow-up rates observed in our study may be reflective of the broader patterns of continuity of care in Australian general practice. The vast majority of Australian patients attend only 1 or 2 general practices annually, and older patients and those with chronic conditions demonstrate particularly high continuity of care [37]. Nevertheless, our findings raise important questions about the representativeness of practices participating in research studies overall. It is possible that these practices are inherently high performing compared to the broader landscape of general practice, potentially skewing our understanding of typical follow-up rates and care quality in primary care settings. Future research needs to explore this issue more thoroughly by comparing the performance metrics of research participating practices with those of a random sample of general practices, investigating the characteristics and motivations of practices that regularly engage in research, and developing strategies to involve a more diverse range of practices in primary care research. Understanding these factors is crucial for accurately assessing the generalizability of research findings and for developing interventions that can be effectively implemented across the entire spectrum of primary care practices. Regardless of the latter, the high rates of investigation and imaging in our cohort (86% had blood tests and 61% had imaging) are consistent and may reflect the accessibility of diagnostic tools in the Australian health care system. This accessibility has been credited for Australia’s comparatively better cancer outcomes relative to similar high-income countries worldwide [35].

The symptom profile of patients with UWL presents an interesting diagnostic challenge. One-third (12/36, 33%) of patients identified by the UWL module had no other recorded symptoms, and nearly half (17/36, 47%) had nonspecific symptoms such as appetite loss and lethargy recorded in any field in the EHR. The high prevalence of no other symptoms or coexisting nonspecific symptoms aligns with international literature and underscores the difficulty in differentiating between benign causes and more serious underlying conditions [8,38,39]. Nicholson et al [39] highlight the complexity of interpreting weight loss in primary care, especially when it cooccurs with other nonspecific symptoms, emphasizing the need for careful clinical assessment and follow-up.

Mental health symptoms were also common in our cohort. Just more than half of the patients received a UWL-related diagnosis, primarily mental health conditions or cancer.

The most prevalent diagnoses in our cohort were mental health conditions, cancer, and chronic obstructive pulmonary disease, consistent with international literature. A study by Withrow et al [6] described the most prevalent serious conditions for people with UWL, stratified by age and sex. Given our cohort’s median age of 68.5 years and equal sex distribution, our findings align with their reported common diagnoses for this age group: cancer, diabetes, and chronic obstructive pulmonary disease in men; and depression, thyroid disorders, and cancer in women.

### Limitations

The algorithm identified 36 (60%) of 60 patients with UWL. Identifying UWL in EHRs is clinically and technically challenging due to inconsistent documentation practices and the predominance of free text in Australian primary care records [40,41]. In this study, “intention” of weight loss was recorded in the clinical notes for 98% of cases. Without access to unstructured data such as the clinical notes, algorithms such as this, or even more sophisticated natural language models and AI applications have very limited potential. Furthermore, if CDSS performance depends wholly on the quality of the recorded symptoms, then if documentation is incomplete, detection will inevitably be affected. Human scribes in primary care have been shown to raise productivity, increase face-to-face time by 39% to 57%, and improve chart quality and physician satisfaction without reducing patient satisfaction [42]. Ambient AI scribes promise to scale this documentation capture, but evaluations show omissions are frequent and occasional high-severity inaccuracies occur, so clinical oversight and careful product selection are essential. Studies in Canada and the United Kingdom reported strong note-generation capabilities across multiple AI scribes but also marked variability in accuracy, usability, and technical performance [43]. Future research should focus on perfecting these technologies. A pragmatic approach would be to layer scribe-captured narratives into CDSS workflows, enabling richer free-text phenotyping (eg, unintentional weight loss with cosymptoms) while preserving clinician control and opportunities for validation, thereby balancing improved capture with safety and trust. The number of false negatives—patients with UWL not identified by the algorithm—also remains unknown, given that we did not review records not flagged by the algorithm due to cost and time constraints. We were therefore only able to identify true positives and false positives identified by the tool.

The algorithm’s failure to exclude 3 patients with cancer (2 cases of lymphoma and myeloma, possibly due to information being solely in clinical notes, and 1 case of breast cancer with a coded diagnosis) highlights an unexpected algorithmic error and underscores the challenges in coding and the need to ensure coding is standardized and reviewed over time. Measured weight loss was not incorporated into the algorithm as an inclusion criterion due to a low percentage of 2 consecutive measurements in preliminary analysis; the impact of this omission on the correct identification of patients remains unclear and warrants further investigation. The study’s small scale, with a limited number of patients, significantly restricts its generalizability. Although relevant and necessary to ensure patient safety and good allocation of resources, larger, more diverse patient cohorts are necessary for more robust and widely applicable findings.

These limitations highlight areas for improvement in future research, emphasizing the need for enhanced data access, refined algorithmic processes, and larger study populations to increase the reliability and applicability of methods for identifying patients with UWL.

## Conclusions

Our study provides valuable insights into the implementation of a CDSS for identifying patients with UWL at risk of cancer in primary care. While we encountered challenges, particularly around tool integration and correct identification of patients with UWL, we also observed high rates of follow-up care and comprehensive investigations in our participating practices. These findings highlight both the potential of CDSS in improving patient care and the complexities involved in their successful implementation. Future research should focus on enhancing CDSS integration with existing workflows, improving access to unstructured data, to ensure clinicians’ trust in these tools, specifically to enhance the usability of digital interventions, and leverage other, more sophisticated digital technologies.

## Supplementary material

10.2196/90885 Multimedia Appendix 1 Interview schedule.

10.2196/90885 Multimedia Appendix 2 Data extraction template.

10.2196/90885 Multimedia Appendix 3 Uunintended weight loss criteria for clinical audits.

10.2196/90885 Multimedia Appendix 4 Summary of themes.

10.2196/90885 Multimedia Appendix 5 Detailed audit table.

10.2196/90885 Checklist 1 COREQ checklist.

## Abbreviations

CDSS: clinical decision support system
COREQ: Consolidated Criteria for Reporting Qualitative
EHR: electronic health record
FHT: Future Health Today
GP: general practitioner
QI: quality improvement
UWL: unintended weight loss

## References

[1] Neal RD, Tharmanathan P, France B (2015). Is increased time to diagnosis and treatment in symptomatic cancer associated with poorer outcomes? Systematic review. Br J Cancer.

[2] Hamilton W, Walter FM, Rubin G, Neal RD (2016). Improving early diagnosis of symptomatic cancer. Nat Rev Clin Oncol.

[3] Smith TJ, Hillner BE (2011). Bending the cost curve in cancer care. N Engl J Med.

[4] Britt H (2016). General Practice Activity in Australia 2015-16.

[5] Primary health care. World Health Organization.

[6] Withrow DR, Oke J, Friedemann Smith C, Hobbs R, Nicholson BD (2022). Serious disease risk among patients with unexpected weight loss: a matched cohort of over 70 000 primary care presentations. J Cachexia Sarcopenia Muscle.

[7] Nicholson BD, Hamilton W, Koshiaris C, Oke JL, Hobbs FDR, Aveyard P (2020). The association between unexpected weight loss and cancer diagnosis in primary care: a matched cohort analysis of 65,000 presentations. Br J Cancer.

[8] Hernández JL, Riancho JA, Matorras P, González-Macías J (2003). Clinical evaluation for cancer in patients with involuntary weight loss without specific symptoms. Am J Med.

[9] Hamilton W (2009). The CAPER studies: five case-control studies aimed at identifying and quantifying the risk of cancer in symptomatic primary care patients. Br J Cancer.

[10] Shortliffe EH, Cimino JJ (2014). Biomedical Informatics: Computer Applications in Health Care and Biomedicine.

[11] Chima S, Reece JC, Milley K, Milton S, McIntosh JG, Emery JD (2019). Decision support tools to improve cancer diagnostic decision making in primary care: a systematic review. Br J Gen Pract.

[12] Price S, Spencer A, Medina-Lara A, Hamilton W (2019). Availability and use of cancer decision-support tools: a cross-sectional survey of UK primary care. Br J Gen Pract.

[13] Chima S, Hunter B, Martinez-Gutierrez J (2025). Implementation of a quality improvement and clinical decision support tool for cancer diagnosis in primary care: process evaluation. JMIR Cancer.

[14] May CR, Mair FS, Dowrick CF, Finch TL (2007). Process evaluation for complex interventions in primary care: understanding trials using the normalization process model. BMC Fam Pract.

[15] Hunter B, Biezen R, Alexander K (2020). Future Health Today: codesign of an electronic chronic disease quality improvement tool for use in general practice using a service design approach. BMJ Open.

[16] Chima S, Martinez-Gutierrez J, Hunter B, Manski-Nankervis JA, Emery J (2022). Optimization of a quality improvement tool for cancer diagnosis in primary care: qualitative study. JMIR Form Res.

[17] Chima S, Martinez-Gutierrez J, Hunter B (2025). Future Health Today and patients at risk of undiagnosed cancer: a pragmatic cluster randomised trial of quality- improvement activities in general practice. Br J Gen Pract.

[18] Hunter B, Alexander K, Biezen R (2023). The development of Future Health Today: piloting a new platform for identification and management of chronic disease in general practice. Aust J Prim Health.

[19] Lee A, de Mendonça L, McCarthy D (2025). Primary care patients presenting with unexpected weight loss in Australian general practices: replication of a diagnostic accuracy study. BMJ Open.

[20] Practice incentives program quality improvement measures: annual data update 2024–25, QIM 3: height and weight recorded and BMI. Australian Institute of Health and Welfare.

[21] Martinez-Gutierrez J, De Mendonca L, Ly P (2024). A scoping review of unexpected weight loss and cancer: risk, guidelines, and recommendations for follow-up in primary care. BJGP Open.

[22] Singh H, Sittig DF (2020). A sociotechnical framework for safety-related electronic health record research reporting: the safer reporting framework. Ann Intern Med.

[23] Van de Velde S, Kunnamo I, Roshanov P (2018). The GUIDES checklist: development of a tool to improve the successful use of guideline-based computerised clinical decision support. Implement Sci.

[24] Harris PA, Taylor R, Thielke R, Payne J, Gonzalez N, Conde JG (2009). Research electronic data capture (REDCap)--a metadata-driven methodology and workflow process for providing translational research informatics support. J Biomed Inform.

[25] Braun V, Clarke V (2012). APA Handbook of Research Methods in Psychology.

[26] Sekhon M, Cartwright M, Francis JJ (2017). Acceptability of healthcare interventions: an overview of reviews and development of a theoretical framework. BMC Health Serv Res.

[27] (2025). Modified monash model. Australian Government Department of Health, Disability and Ageing.

[28] Ancker JS, Edwards A, Nosal S (2017). Effects of workload, work complexity, and repeated alerts on alert fatigue in a clinical decision support system. BMC Med Inform Decis Mak.

[29] Carayon P, Schoofs Hundt A, Karsh BT (2006). Work system design for patient safety: the SEIPS model. Qual Saf Health Care.

[30] May C, Finch T (2009). Implementing, embedding, and integrating practices: an outline of Normalization Process Theory. Sociology.

[31] Proctor EK, Powell BJ, McMillen JC (2013). Implementation strategies: recommendations for specifying and reporting. Implement Sci.

[32] Kortteisto T, Komulainen J, Mäkelä M, Kunnamo I, Kaila M (2012). Clinical decision support must be useful, functional is not enough: a qualitative study of computer-based clinical decision support in primary care. BMC Health Serv Res.

[33] Cabana MD, Rand CS, Powe NR (1999). Why don’t physicians follow clinical practice guidelines? A framework for improvement. JAMA.

[34] Lynch C, Harrison S, Emery JD (2023). Variation in suspected cancer referral pathways in primary care: comparative analysis across the International Benchmarking Cancer Partnership. Br J Gen Pract.

[35] Lacey K, Bishop JF, Cross HL, Chondros P, Lyratzopoulos G, Emery JD (2016). Presentations to general practice before a cancer diagnosis in Victoria: a cross-sectional survey. Med J Aust.

[36] Wright M, Hall J, van Gool K, Haas M (2018). How common is multiple general practice attendance in Australia?. Aust J Gen Pract.

[37] Nicholson BD, Perera R, Thompson MJ (2018). The elusive diagnosis of cancer: testing times. Br J Gen Pract.

[38] Nicholson BD, Hamilton W, O’Sullivan J, Aveyard P, Hobbs FR (2018). Weight loss as a predictor of cancer in primary care: a systematic review and meta-analysis. Br J Gen Pract.

[39] Nicholson BD, Aveyard P, Hamilton W, Hobbs FDR (2019). When should unexpected weight loss warrant further investigation to exclude cancer?. BMJ.

[40] Canaway R, Boyle D, Manski-Nankervis JA, Gray K (2022). Identifying primary care datasets and perspectives on their secondary use: a survey of Australian data users and custodians. BMC Med Inform Decis Mak.

[41] Zallman L, Finnegan K, Roll D, Todaro M, Oneiz R, Sayah A (2018). Impact of medical scribes in primary care on productivity, face-to-face time, and patient comfort. J Am Board Fam Med.

[42] Ha E, Choon-Kon-Yune I, Murray L (2025). Evaluating the usability, technical performance, and accuracy of artificial intelligence scribes for primary care: competitive analysis. JMIR Hum Factors.

[43] Draper TC, Cox T, Lamb-Riddell K (2025). Clinical AI Scribes in primary care: accuracy, error severity and implications for clinical practice. BMJ Digit Health.

