# Supplementary material for: Piloting a Clinical Decision Support System for Unintended Weight Loss in Primary Care: Mixed Methods Study on Early Cancer Detection
Source: JMIR Cancer. 2026 Jul 28;12:e90885. doi: 10.2196/90885 (PMC13411435; doi:10.2196/90885)
Supplement: Multimedia Appendix 1 [file cancer-v12-e90885-s001.docx]

# **Semi-structured interview guide:**

## Interviews with general practice staff

## Notes

- Questions may vary depending on who is being interviewed – a GP or someone from the cohort review team (e.g., a PN overseen by a GP)

## Interview guide for GP

**Introductions**

**Age**

**Gender**

**Practice role**

**Years in practice**

**Role in UWL module**

## CDS system

***Opening questions about the FHT technology generally***

1. Have you been using the Future Health Today tool?

- *If yes, how frequently do you use Future Health Today tool?*
- *How long have you had FHT in your practice?*

1. What parts of the tool have you been using? (portal/PoC)
2. What do you like the most about the tool?
3. What do you like the least about the tool?

## CDS implementation

1. Were you involved in the cohort review process? Can you tell me about the cohort review process?

- *Did you recall any patients? Can you describe this process?*
- *How did you manage the cohort review? Who was involved?*

1. Did you see patients after they were recalled?

- *Did you see the recommendation/how easy was it to find the recommendation to understand why the patient was recalled*

## CDS content

1. Have you seen any UWL recommendations at the point of care/in the EMR during a patient consultation?

- *Can you tell me about the recommendations you have seen relating to unexpected weight loss and/or cancer risk?*
- *Have you had a cancer risk recommendation either pop up with a patient or when you review of their files?*
- *Did you have to scroll to see the recommendation?*

1. What are your initial thoughts regarding the recommendations?

*Potential probing questions:*

- *How clear are they?*
- *Did you find any of the recommendations surprising?*
- *Have you queried or felt like you need more information to understand any of the recommendations?*
- *How confident were you in your ability to use and understand the FHT recommendations? Is there anything that would make you feel more comfortable?*

1. How **useful** do you find the recommendations?

*Potential probing questions:*

- *In the context of your everyday practice, how useful do you think the recommendations are for identifying people at risk of cancer?*
- *Have the cancer recommendations influenced/changed your understanding and management of patients with unexpected weight loss? (if yes, how? In what ways?)*

## CDS context

1. Have you used the UWL recommendation in a consultation with a patient?

*Potential probing questions:*

- *If so, how did the consultation go? If not, why?*
- *How did it affect the workflow?*

1. Do you think the recommendations and recommended actions are **feasible** and what impact would they/do they have on your workflow?

*Potential probing questions:*

- *How do you see the management of these recommendations fitting into usual practice?*

## Interview guide for review team if not a GP

**Introductions**

**Age**

**Gender**

**Practice role**

**Years in practice**

**Role in UWL module**

## CDS system

***Opening questions about the FHT technology generally***

1. Have you been using the Future Health Today tool?

- *If yes, how frequently do you use Future Health Today tool?*
- *How long have you had FHT in your practice?*

1. What parts of the tool have you been using?

- *Tell me about using the portal/PoC*

1. What do you like the most about the tool?
2. What do you like the least about the tool?

## CDS implementation

1. Can you tell me about the cohort review process?

- *Did you review the cohort independently or with a GP?*
- *How did you manage the cohort review? Who was involved?*

1. Did you recall any patients? Can you describe this process?
2. Did you copy the UWL recommendation into the patients file?

- *Did you take any extra steps in communicating the recommendation to the GP during the cohort review process?*

## CDS content

1. What are your initial thoughts regarding the recommendations
   1. *How clear are they?*
   2. *Did you find any of the recommendations surprising?*
   3. *How confident were you in your ability to use and understand the FHT recommendations?*
   4. *Were you able to understand why patients were flagged?*
2. Have you seen any UWL recommendations at the point of care/in the EMR during a patient consultation?

- *Can you tell me about the recommendations you have seen relating to unexpected weight loss and/or cancer risk?*
- *Have you had a cancer risk recommendation either pop up with a patient or when you review of their files?*
- *Did you have to scroll to see the recommendation?*

1. How useful do you find the recommendations?

*Potential probing questions:*

- *In the context of your everyday practice, how useful do you think the recommendations are for identifying people at risk of cancer?*
- *Do you think the recommendations and recommended actions are feasible to implement?*

## CDS context

1. Did you see patients after they were recalled?

- *If so, how did the consultation go? If not, why?*
- *How did it affect the workflow?*

1. Do you think the recommendations and recommended actions are feasible and what impact would they/do they have on your workflow?

*Potential probing questions:*

- *How do you see the management of these recommendations fitting into usual practice?*
