# Supplementary material for: Piloting a Clinical Decision Support System for Unintended Weight Loss in Primary Care: Mixed Methods Study on Early Cancer Detection
Source: JMIR Cancer. 2026 Jul 28;12:e90885. doi: 10.2196/90885 (PMC13411435; doi:10.2196/90885)
Supplement: Multimedia Appendix 2 [file cancer-v12-e90885-s002.pdf]

Clinical fidelity Study

Record ID ______________________

Today's date (date of the audit) ______________________

Practice information

Practice Number
- ◯ 1-Practice 1
- ◯ 2-Practice 2
- ◯ 3-Practice 3
- ◯ 4-Practice 4
- ◯ 5-Practice 5
- ◯ 6-Practice 6

Patient number ______________________
(Assigned by extractor in order of extraction)

What electronic medical record does the practice use?
- ◯ Best Practice
- ◯ Medical director

Information from FHT

Was the patient recalled or deferred?
- ◯ Recalled
- ◯ Deferred
- ◯ Other

If the patient was deferred, what was the reason? ______________________

If other, explain why patient was not recalled or deferred? ______________________

Date of cohort review ______________________

Patient information (from EMR)

Date of Birth ______________________

Age at audit date ______________________
(Age today)

Age at index date ______________________
(This will be calculated once index date is entered in the next section)

Birth Sex	◯ Female<br>◯ Male<br>◯ Missing<br>(Biological Designated Sex)

Unexpected Weight Loss related information

Which recommendation(s) did the patient have?	☐ 6.1b - "Unexpected weight loss detected: In younger men and women (aged < 60 years), prioritise screening for depression and testing for thyroid function and diabetes."<br>☐ 6.1c - "Unexpected weight loss detected: In men aged 60-79, prioritise cancer investigation and testing for diabetes and chronic obstructive pulmonary disorder (especially in smokers)."<br>☐ 6.1d - "Unexpected weight loss detected: In women aged 60-79, prioritise testing for thyroid function and screening for depression while considering cancer investigation."<br>☐ 6.1e - "Unexpected weight loss detected: In older patients (aged 80 and over), prioritise screening for dementia, depression, and heart failure during workup for cancer investigation."<br>☐ 6.2 - "Unexpected weight loss detected: Consider further relevant history and investigations. Recommend physical examination including new weight measurement."<br>☐ 6.3 - "Unexpected weight loss and abnormal test results. Consider increased risk of cancer as differential diagnosis and investigations such as iFOBT, CXR, upper/lower endoscopy, abdominal CT scan and CA_125 (if female)."<br>☐ No recommendation at time of audit
Is there a record of unexpected weight loss?	◯ Yes<br>◯ No
What was the index date of the UWL record?	__________________________________
Has the patient been seen since the UWL index date?	◯ Yes<br>◯ No
If yes, how many times?	__________________________________
When were they seen?	◯ Since index date<br>◯ Since cohort review<br>◯ Both
How many times has the patient been seen post cohort review?	__________________________________
Date of first appointment after cohort review	__________________________________
Have any appointments post cohort review included unexpected weight loss follow-up?	◯ Yes<br>◯ No

If yes, describe. ______________________________________

Intention

Does the reason for encounter/clinical notes provide any information around whether the weight loss was intentional or unintentional?
- ◯ Yes
- ◯ No

What was the reason?
- ◯ Intentional weight loss
- ◯ Unintentional weight loss

Where is the intention or unintention recorded?
- ☐ Reason for encounter
- ☐ Diagnosis
- ☐ Clinical notes
- ☐ Other

If other, describe ______________________________

What does the UWL record say? ______________________________
(Cut and paste text)

Any additional comments about intention? ______________________________
(Any comments to add about intention)

UWL symptom after index date

Was UWL recorded or discussed again after index date?
- ◯ Yes
- ◯ No

Where was it recorded?
- ☐ Reason for encounter
- ☐ Diagnosis
- ☐ Clinical notes
- ☐ Other

If other, describe ______________________________

What does it say? ______________________________
(Cut and paste text)

Any additional comments about UWL? ______________________________

Pathologies

Have any investigations been ordered since the index date?

- ◯ Yes
- ◯ No

When were investigations ordered?

- ◯ Since index date
- ◯ Since cohort review
- ◯ Both

What investigations have been ordered?

- ☐ Liver Function (Albumin + ALP + Bilirrubin)
- ☐ C Reactive Protein (CRP)
- ☐ ESR
- ☐ Calcium
- ☐ Creatinine
- ☐ Full Blood Count
- ☐ FOBT
- ☐ Ca125
- ☐ TSH
- ☐ HBA1C
- ☐ Fasting Blood glucose
- ☐ HIV
- ☐ Dementia screening
- ☐ Urinalysis
- ☐ Urine culture
- ☐ Vitamin b12
- ☐ Iron studies
- ☐ Other

If other, describe

____

When were the first investigation(s) ordered?

____

Date of subsequent investigations

____

Any additional comments about investigations?

____

Referrals and imaging

Was the patient referred for further investigation?

- ◯ Yes
- ◯ No

To which specialty was the patient referred?

- ☐ Oncology
- ☐ Haematology
- ☐ Pneumology
- ☐ Gastroenterology
- ☐ Nephrology
- ☐ Gynecologist
- ☐ Dietician
- ☐ Other

If other, describe

____

Date of first referral

____

Date of subsequent referral

____

Any additional comments about referrals?

____

What imaging exams were ordered after the index date?

- ☐ None
- ☐ CT scan
- ☐ MRI
- ☐ X-ray
- ☐ Ultrasound
- ☐ Colonoscopy
- ☐ Endoscopy
- ☐ Other
- ☐ Echocardiography

(Exams ordered AFTER index date)

If other, describe

____

When were they ordered?

- ◯ Since index date
- ◯ Since review
- ◯ Both

Any additional comments about imaging?

____

Diagnoses

Has a diagnosis been made after the index date that might be related to UWL?

- ◯ Yes
- ◯ No

When was the diagnosis made?

- ◯ Since index date
- ◯ Since review
- ◯ Both

Select all diagnoses since index date

- ☐ Cancer
- ☐ Diabetes
- ☐ COPD
- ☐ Depression
- ☐ Thyroid disorders
- ☐ Heart Failure
- ☐ Dementia
- ☐ Rheumatoid Arthritis
- ☐ Alcohol Addiction
- ☐ Eating disorders
- ☐ Malabosorption
- ☐ Irritable Bowel Disease
- ☐ other

If other, describe

____

Select all diagnoses since cohort review	☐ Cancer<br>☐ Diabetes<br>☐ COPD<br>☐ Depression<br>☐ Thyroid disorders<br>☐ Heart Failure<br>☐ Dementia<br>☐ Rheumatoid Arthritis<br>☐ Alcohol Addiction<br>☐ Eating disorders<br>☐ Malabosorption<br>☐ Irritable Bowel Disease<br>☐ other
If other, describe	__________________________________
Which cancer was diagnosed?	__________________________________
Date of first diagnosis	__________________________________
Date of subsequent diagnosis	__________________________________
Where is the diagnosis/diagnoses recorded?	☐ Reason for encounter<br>☐ Diagnosis<br>☐ Clinical notes<br>☐ Other
If other, describe	__________________________________
Any additional comments about diagnosis?	__________________________________

Symptoms/presentations

Were any other symptoms recorded at index date?	◯ Yes<br>◯ No
What other symptoms were recorded at index date?	__________________________________
Were there other symptoms described in association with UWL in subsequent consultations?	◯ Yes<br>◯ No
What symptoms were described in subsequent consultations?	__________________________________
When were subsequent symptoms recorded?	◯ Since index date<br>◯ Since cohort review<br>◯ Both

Where are the symptoms recorded?	☐ Reason for encounter<br>☐ Diagnosis<br>☐ Clinical notes<br>☐ Other
If other, describe	__________________________________
Any additional comments about symptoms?	__________________________________

Lifestyle information

Smoking Status	◯ Smoker<br>◯ Ex-Smoker<br>◯ Non smoker<br>◯ Not completed<br>(most recently recorded)
Cigarettes per day	__________________________________
Any additional comments about smoking habits?	__________________________________<br>(any smoking related information)
Drinking Status	◯ Current<br>◯ Non drinker<br>◯ Missing<br>(Most recently recorded)
Current alcohol intake - days per week	__________________________________
Current alcohol intake - standard drinks per day	__________________________________
Any additional comments about alcohol intake?	__________________________________

Weight measurements

Does the patient have a weight measurement recorded at index date?	◯ Yes<br>◯ No
Weight measurement at index date?	__________________________________<br>(kg)
Recorded weight loss?	◯ Yes<br>◯ No
Amount of weight lost	__________________________________<br>(kgs)

Does the patient have another weight measurement after index date?	◯ Yes<br>◯ No
Most recent weight measurement	____________________<br>(kg)
Current weight loss?	◯ Yes<br>◯ No
Amount of weight lost	____________________<br>(kgs)
Does the patient have a recorded BMI on or after the index date?	◯ Yes<br>◯ No
What is the BMI?	____________________
When was it recorded?	____________________
Any other BMI comments?	________________________
