# Supplementary material for: Piloting a Clinical Decision Support System for Unintended Weight Loss in Primary Care: Mixed Methods Study on Early Cancer Detection
Source: JMIR Cancer. 2026 Jul 28;12:e90885. doi: 10.2196/90885 (PMC13411435; doi:10.2196/90885)
Supplement: Multimedia Appendix 3 [file cancer-v12-e90885-s003.docx]

**UWL criteria for clinical audits**

| **Outcome** | **Definition** |
| --- | --- |
| True UWL (correct classification) | The algorithm's diagnosis of UWL matched the researchers' clinical assessment based on EHR data analysis. |
| False UWL (incorrect classification) | The algorithm's diagnosis of UWL did not match the researchers' clinical assessment based on EHR data analysis. |
| Inconclusive UWL (unable to classify) | The algorithm flagged UWL, but EHR data was insufficient to confirm if the weight loss was intentional or not. |

Clinician’s case classification criteria for true UWL:

1. EHR contains synonyms for "unintended weight loss" (unexpected, not intentional, does not want to lose weight etc)
2. GP concern about weight loss evidenced by:
   - Diagnostic tests ordered
   - Referrals sent
   - Follow-up appointments scheduled
   - Watchful waiting documented
   - Patient concern noted
3. No mentions of intentional weight loss or related medications (weight loss programme, wants to lose weight, weight lose advice etc)

Algorithm accuracy was calculated as = (True UWL cases) / (Total audited files)
