# Supplementary material for: Piloting a Clinical Decision Support System for Unintended Weight Loss in Primary Care: Mixed Methods Study on Early Cancer Detection
Source: JMIR Cancer. 2026 Jul 28;12:e90885. doi: 10.2196/90885 (PMC13411435; doi:10.2196/90885)
Supplement: Multimedia Appendix 4 [file cancer-v12-e90885-s004.docx]

Summary of themes and relevant quotes

| **Themes/Dimensions** | **Summary** | **Quotes** |
| --- | --- | --- |
| **Socio-technical model** | | |
| Clinical content | Staff generally found the content clear and appropriate, though many admitted not paying close attention due to their focus on audits. Some confusion arose regarding the dual recommendations for UWL, with one practice viewing it as duplication. A practice owner noted potential difficulties in addressing differential diagnoses not on the patient's agenda. While experienced practices found recommendations easy to understand and use, most users didn't fully grasp the flagging criteria. Despite these issues, the tool was appreciated as a reminder system for various diagnoses, not just cancer. | “Yeah, they're both for unintended weight loss. And I do, I do get the clinical context is slightly different for patients of advanced age. (…) but I think the recommendations themselves were quite straightforward and certainly consistent with what I would expect would be usual or best practice. Practice pharmacist/QI, Practice 1  “I must admit, I didn't closely look at that. I more went to look at their weight records, first of all, to see, to see the veracity of the concern about their weight loss.” Practice 2.  (…) “it actually says here, prioritize screening for depression, right? But if the patient presentation is for, I don't know, they've got the flu today, or they've got COVID. It's going to be a very difficult conversation for the doctor.” Practice 5  “It's probably reminded me that it is something we need to look at, particularly in older individuals who might be losing weight for a variety of reasons, particularly if they're not, you know, mobile, but even if there's no underlying issue with malignancy or depression or heart failure, you know, are they losing weight because they're sarcopenic, and then we've got an increased falls risk that we've got to consider. So it's probably been a reminder to not just accept weight loss as a natural part of aging, if that makes sense.” Practice 1 |
| External rules, regulations and pressures | Two practices highlighted concerns about potential false positives due to increased interest in weight loss and new weight loss medications. GPs were discouraged from recording weight loss as a reason for visit in the EHR due to the lack of distinction between intended and unintended weight loss, which limited its clinical relevance. Additionally, existing incentives for other preventive activities may have competed for attention with the UWL recommendations. | “That may be to do just with timing, because this was all running while (weight loss drug) was launched, and there was a lot of buzz in the community about weight loss medications” Practice 1.  “I rarely tick weight loss as a diet as a reason for visit because, because it is rather meaningless without some context” Practice 2  So at the moment, PN is working on the diabetes register to call patients back for their chronic disease management plans for an ABI (Ankle brachial index). So, she's just working through (practice EHR).Practice 4 |
| Hardware and software computing infrastructure | Four practices identified the lack of integration of the cohort tool into existing software as a significant barrier to implementation, while the integrated pop-up was seen as more feasible. Limited software capability to identify patients beyond coded fields was also noted as an obstacle. One GP reflected on their EHR software's ability to distinguish between unintended and intentional weight loss. Additionally, real-time data capability was highlighted as a desirable feature for the FHT software, suggesting a need for more dynamic and responsive tools. | “(..) but then you've still got to go and do stuff in patient records as well, if you are flagging people for recall” Practice 1  “Oh, feasibility. It's entirely feasible to have to have the pop up running” Practice 2  “Yeah, it because it could be like, lost 20 kilos without trying. But reason for visit sore knees. You know what I mean? Like, that's that person that's got 20 kilos of weight loss, unintended won't come into the cohort.” Practice 3  “And so maybe if there was a distinction between, you know, patient concern about unexplained weight loss, versus patients coming in seeking weight loss and the at the moment, the available options in our software don't allow us to distinguish that. And yeah, and for a whole range of reasons, I am reluctant to tick a box saying, weight loss” Practice 2  “That's the one of the big advantages of (our EHR). It's real time data, whereas, with a lot of the other add ons that we've had, (…) they're not necessarily real time. So it makes it a bit hard when you're extracting data that today's data might not be relevant to two weeks later.” Practice 4 |
| Human computer interface | Implementation and usage of the platform varied significantly among practices. Three clinicians didn't use it at all, relying on provided patient lists instead. One practice effectively utilised the defer and recall tools for chronic management, though recall required external software. They preferred deferring directly from the EHR rather than the cohort tool. A staff member suggested displaying all relevant recommendations simultaneously alongside the EHR for better visibility. Integration issues were noted, particularly regarding the patient recall component's compatibility with Best Practice software, highlighting the need for improved functionality and seamless integration with existing systems. | “But I would imagine if it popped, if it popped automatically along the side of the file, but not take up much room, but not take up much room.” Practice 3  “I find the fact that once you've flagged it for recall, it kind of stops. It doesn't talk back to best practice to add any reminders or add any actions, … so it does feel kind of like a fragmented approach in you know, you're doing all this in FHT, but then you've still got to go and do stuff in patient records as well, if you are flagging people for recall.” – Practice 1 (could also be part of workflow) |
| People | he tool's appeal and effectiveness may vary depending on the user's role and interests. Those focused on quality improvement might find it more beneficial compared to clinicians. A significant barrier noted was the lack of allocated time for GPs to engage with the tool. Some practices reported that doctors were less involved or harder to engage with the system, leading to the development of internal procedures to involve GPs where necessary. This suggests a need for strategies to better integrate the tool into GP workflows and potentially tailor its features to different user groups within the practice. | “But if I put a list of patient names (for follow-up) on a GPS desk, it's going to have disappeared into a black hole within 15 minutes.” Practice 1  “I mean, I in general, I like quality improvement anyway, so I always like to - both PM and I really like data” Practice 1  “No, no, I haven't got it on for the GPs at all. And the reason for that is most of my doctors are anti pop ups” Practice 5.  “…the hardest, probably part, is to get the doctors involved as such, they are happy for us to do the work though, and then, you know, bring it to their attention and stuff like that. But no, they don't want to do it too much.” Practice 3 |
| System measurement and monitoring | Clinical review of the model was deemed essential due to perceived inaccuracies in patient identification. Confusion arose regarding inclusion criteria, with the model seemingly flagging patients with intentional weight loss, no actual weight loss, or existing cancer diagnoses. One practice expressed concern that the system might miss relevant patients due to doctors' use of free text in clinical notes, which the algorithm couldn't process. Data entry errors were reported to cause false positives in one practice. The same practice also noted algorithm issues, such as incorrectly including patients with known cancer diagnoses who should have been excluded. These observations highlight the need for refinement in the model's accuracy and its ability to interpret various data entry formats. | “There were several who were under investigation for likely cancer, but that was already in process. And there were others who had lost weight for other medical reasons with exacerbation of heart failure or COPD or other medical issues” Practice 2  “And I also believe that the cohort would be way bigger if it was coded in our clinical software. So people might come in for that, but the doctor hasn't coded it as that (…) Some doctors don't do a reason for visits” Practice 3 |
| **Theoretical framework of acceptability** | | |
| Burden | The cohort tool's burden varied among staff roles and practices. Practice Managers (PMs) and Practice Nurses (PNs) found it manageable due to small cohort sizes, while GPs were less likely to use it. The pop-up feature was considered burdensome for GPs, leading two practices to avoid installing it on GP computers. Practices 4 and 5, under the same PM, preferred using their EHR for queries, viewing FHT as an extra step. The small cohort size was crucial in keeping the burden low, though follow-up with doctors for patient scheduling was still necessary. Despite false positives, Practice One saw long-term value in the tool due to the low number of patients to review. Overall, the smaller cohorts associated with this module enhanced its acceptability by reducing the workload. | “Yeah, yeah. But it's just in that sense, it's just yet another extra, extra task for GPs to do. You know, in our family time unpaid, and if it's useful, then GPs are generally happy to do that. But really like to see some, some tangible benefit.” Practice 2  “That with the unintended weight loss, it wasn't too bad, because with only a small cohort, so it was easier to go through here” Practice 3  “It's just built into it (EHR query tool). So, you know, she's just got to do two clicks and then she can quickly have a look into the patient file. Whereas with FHT, you know, because it's an outside model, she actually had to sit in this room where the FHT was, log into the best practice and then look through two different ones, then cut through the data, because it only took certain information. So, you know, I had to allocate a good hour. Fortunately, there wasn't that many patients, but if there were more patients, she'd (PN) need more time for it.” Practice 4  “I think it's still useful, because you're not dealing with a lot of patients in total, like we had 11 over the course of the few months that we were looking at it.” – Practice 1  “I guess the downside is that there's often limited time in a consultation, and it's a matter of time management, and if it pops up, you know, several different messages for a patient about their risk of various things, and none of which have got anything to do with the reason they came in to see me. It's very difficult to cover all of that in time, and ideally we would get the patient to come back and go through some of those things.” Practice 2 |
| Ethicality | One practice mentioned patient recall for delayed appointments may cause unnecessary stress and alarm. | “If it's clicking up a concern about something that we don't want to alarm the patient, and it can be difficult sometimes to explain to the patient why we want them to come back, and sometimes patients are sceptical of our motives for calling them back and also, you know, like my appointments are booked out for weeks and weeks ahead, so it's really hard to fit patients in in a timely fashion.” Practice 2 |
| Opportunity costs | The tool's perceived value varied among staff and practices. A Practice Nurse found reviewing the short list manageable and potentially beneficial for identifying overlooked UWL cases. However, scheduling delays were seen as a drawback. For patients, the concern was that addressing UWL might overshadow their original reasons for visiting, potentially requiring additional appointments. One practice was considering installing the tool on nurses' computers for review but expressed concerns about system performance and alignment with their preventive objectives. They also noted that using their own EHR system for preventive management seemed more straightforward, highlighting the importance of seamless integration and efficiency in adopting new tools. | “I think it's still useful, because you're not dealing with a lot of patients in total, like we had 11 over the course of the few months that we were looking at it. So even though I had to look at every patient file that didn't really take a lot of time, and we've got three patients that may potentially have an issue that's been undiagnosed, so I think the benefits are still going to outweigh the time that you've taken in scrutinizing all those records” *Practice 1*  “Like my appointments are booked out for weeks and weeks ahead, so it's really hard to fit patients in in a timely fashion”.  *Practice 2*  “But you know, the nurses will often do a lot of preventative health searches. So at the moment, they were focusing on diabetes and just calling patients back for chronic disease management plans, for flu vaccines, for COVID vaccines. Well, we've got stock, so they're quite proactive in that, in that way.”  *Practice 5* |
| Perceived effectiveness | While most practice staff viewed the model positively in theory, its effectiveness was difficult to assess due to the small number of patients identified. Four practices reported that most flagged patients were already being followed up, though one practice appreciated recalling three overlooked patients. The recommendations were often irrelevant, particularly for cases of intentional weight loss. Patient recall proved challenging, especially if patients didn't understand the reason. Conversely, some expected patients weren't flagged, possibly due to coding issues. Opinions on the tool's utility varied, with some seeing potential value after modifications (such as refining recommendations and reducing false positives), while others found it unhelpful. Despite the clinical importance of the message, GPs tended to ignore it, suggesting a need for improved engagement strategies. | “I think people would generally seem to be enthusiastic about the concept, yeah, but probably not enough information from a single patient age for them to give much meaningful feedback.” Practice 2  So even though I had to look at every patient file that didn't really take a lot of time, and we've got three patients that may potentially have an issue that's been undiagnosed, so I think the benefits are still going to outweigh the time that you've taken in scrutinizing all those records” X Practice 1  “But I think it was more like I did a rough estimate, like 55% were unintended and 45 were intended weight loss, and a lot of them had had lots of investigations, and they've all been normal, but they're just monitoring again, so we've just deferred them for a short like, six months’ time to review again’ Practice 3  “But sometimes patients are reluctant to come back unless you give a clear explanation of why, understandably, and that can be not a straightforward process. Practice 2  “There were several who were under investigation for likely cancer, but that was already in process.” – Interview 2  “I did I did note that I could think off the top of my head of a small number of patients of mine who might been concerned about unexplained weight loss, who were not flagged with this and I wondered why they weren't picked up.” – Interview 2  “I mean, it's good clinical information, but the doctors just have started to ignore that information, so they just turn it off.” – Interview 4 |
| Self-efficacy | The cohort tool's usability varied based on users' prior experience with FHT. Most interviewees, especially those familiar with FHT, found it easy to use. Clinical personnel conducted EHR audits to assess UWL, which they also found straightforward. However, in two practices, nurses were unaware of the tool as Practice Managers sent them spreadsheets for review, while doctors didn't use the cohort tool at all. The level of FHT expertise significantly influenced tool usage mechanisms and user confidence. All practices had previous FHT experience from trials, but those with more extensive experience had established processes in place, highlighting the importance of familiarity and training in effective implementation. | “Yeah, she took the reins on this one because I found it to be - I wasn't confident to look in there I guess and to make the decision on whether it was unintended or not, or I can do the process of the investigations that are ordered. But I, yeah, I didn't.” Practice 3  “I didn't find it too hard. It was - once you went into the patient's file it was pretty self explanatory if you just kept going through the flow of the appointments and what the doctors - because you can see where it said weight loss in the reason for visit, if you sort of read from then on, and then go back if you have to, but, it sort of flowed pretty well, so you could understand.” Practice 3  “I think it was pretty easy to use. Don't really have any complaints” Practice 4  I'm okay with it now, yeah, just knowing, knowing what to do. But I like, I trust it now. I trust myself with it now, like, I don't mind using the point of care tool and deferring people and stuff like that, if I know that they've had it actioned.” Practice 3  “But I used - as part of actmed I used it quite a lot. We were one of the pilot sites here, so I did a lot of that, and then I actually did some of the additional training for the other ActMed pharmacists when they came on board in how to use FHT, so I feel relatively confident.” Practice 1 |
