# Supplementary material for: Piloting a Clinical Decision Support System for Unintended Weight Loss in Primary Care: Mixed Methods Study on Early Cancer Detection
Source: JMIR Cancer. 2026 Jul 28;12:e90885. doi: 10.2196/90885 (PMC13411435; doi:10.2196/90885)
Supplement: Multimedia Appendix 5 [file cancer-v12-e90885-s005.docx]

# Detailed clinical characteristics and follow-up of patients with confirmed unintentional weight loss (N: 36 patients)

|  |  | N (%) |
| --- | --- | --- |
| Number of visits after initial UWL visit (6 months follow up period) | Median (range) | 8.5 (0-41) |
| UWL discussed again after initial UWL visit (6-month follow-up period) | Yes | 20 (56) |
|  | No | 16 (44) |
| Number of patients followed up after initial UWL presentation?^a^ |  | 34 (94) |
| Other presenting symptoms at index date^b^ | | |
| None |  | 12 (33) |
| Non-Specific | Appetite Loss | 6 (17) |
|  | Dizziness | 1 (3) |
|  | Fatigue/lethargy/malaise | 4 (11) |
|  | Nausea | 4 (11) |
|  | Sweats | 2 (6) |
| Gastrointestinal | Abdominal pain | 2 (6) |
|  | Change of bowel habit | 1 (3) |
|  | Constipation | 3 (8) |
|  | Diarrhoea | 2 (6) |
|  | Dysphagia | 1 (3) |
|  | Epigastric pain | 2 (6) |
|  | Vomiting | 2 (6) |
| Mental health | Stress/anxiety/mood/insomnia | 7 (19) |
| Respiratory | Asthma exacerbation  Cough | 1 (3)  2 (6) |
| Other | Oral symptoms | 2 (6) |
|  | Irregular menstruation | 1 (3) |
|  | Non-GI or unspecified pain | 4 (11) |
|  | Urinary symptoms | 1 (3) |
| Investigations ordered by GP |  |  |
|  | Yes | 31 (86) |
|  | No | 5 (14) |
| Investigation ordered^c^ |  |  |
| Haematology | Full blood count | 30 (83) |
|  | Iron studies | 23 (64) |
| Metabolic | Calcium | 22 (61) |
|  | Creatinine | 25 (69) |
|  | Fasting blood glucose | 8 (22) |
|  | Multiple biochemical analysis | 1 (3) |
|  | Thyroid Function | 18 (50) |
|  | Hba1c | 16 (44) |
| Cancer markers | Ca125 | 2 (6) |
|  | Ca15-3 | 1 (3) |
|  | Ca19-9 | 1 (3) |
|  | CEA | 2 (6) |
|  | BCR-ABL | 1 (3) |
|  | Kappa and lambda free light chains | 1 (3) |
| Coeliac serology | Coeliac Antibodies | 1 (3) |
|  | Coeliac disease | 1 (3) |
| Dementia screening |  | 1 (3) |
| Inflammatory markers | CRP | 14 (39) |
|  | ESR | 14 (39) |
| Sexually transmitted infections | Chlamydia | 2 (6) |
|  | Gonorrhoea | 1 (3) |
|  | HIV | 5 (14) |
|  | Syphilis | 1 (3) |
|  | Treponema | 1 (3) |
| Urinary diagnostic panel | Urinalysis | 12 (33) |
|  | Urine culture | 7 (19) |
| Viral markers | Cytomegalovirus | 2 (6) |
|  | Epstein Barr virus | 1 (3) |
|  | Hepatitis B | 2 (6) |
|  | Rubella | 1 (3) |
|  | Varicella | 1 (3) |
| Vitamins | Folate | 1 (3) |
|  | Vitamin B12 | 10 (28) |
|  | Vitamin D | 4 (11) |
|  | Other | 10 (28) |
| Imaging referral, n (%) |  |  |
|  | Yes | 22 (61) |
|  | No | 14 (39) |
| Imaging test ordered^d^ |  |  |
|  | CT scan | 13 (36) |
|  | Colonoscopy | 5 (14) |
|  | Endoscopy | 5 (14) |
|  | X-ray | 7 (19) |
|  | Ultrasound | 3 (8) |
|  | Other | 2 (6) |
| Referrals |  |  |
|  | Yes | 20 (56) |
|  | No | 16 (44) |
| Referrals (detail)^e^ |  |  |
|  | General surgeon for colonoscopy | 4 (11%) |
|  | General surgeon for gastroscopy | 3 (8%) |
|  | Gastroenterology for endoscopy | 2 (6%) |
|  | Dietician | 5 (14%) |
|  | Urology | 1 (3%) |
|  | Psychology | 1 (3%) |
|  | Cardiology | 2 (6%) |
|  | Oncology | 2 (6%) |
|  | Gynaecology | 1 (3%) |
|  | Hematology | 1 (3%) |
|  | Rheumatology | 1 (3%) |
|  | Other | 2 (6%) |
| Related diagnoses within 6 months of UWL appointment to audit date recorded^f^ |  |  |
|  | Yes | 20 (56%) |
|  | No | 16 (44%) |
| Comorbidities^g^ |  |  |
|  | Mental health conditions | 7 (19%) |
|  | Cancer | 4 (11%) |
|  | Chronic Obstructive Pulmonary Disease (COPD) | 3 (8%) |
|  | Gastrointestinal conditions | 2 (6%) |
|  | Musculoskeletal conditions | 2 (6%) |
|  | Frailty | 1 (3%) |
|  | Cognitive/memory impairment | 1 (3%) |
|  | Diabetes | 1 (3%) |
|  | Chronic kidney disease | 1 (3%) |
|  | Parathyroid adenoma | 1 (3%) |
