# Supplementary material for: Piloting a Clinical Decision Support System for Unintended Weight Loss in Primary Care: Mixed Methods Study on Early Cancer Detection
Source: JMIR Cancer. 2026 Jul 28;12:e90885. doi: 10.2196/90885 (PMC13411435; doi:10.2196/90885)
Supplement: Checklist 1 [file cancer-v12-e90885-s006.pdf]

# Coreq Checklist.

| | | | Reported? |
|---|---|---|---|
| **Domain 1: Research team and reflexivity** | | | |
| Personal Characteristics | | | |
| 1. | Interviewer/facilitator | Which author/s conducted the interview or focus group? | Page 7, Line 172: "JMG conducted the interviews." |
| 2. | Credentials | What were the researcher's credentials? *E.g. PhD, MD* | Page 7, Line 174: "JMG is a female mid-career researcher and academic GP with experience in qualitative research. SC is an early-career researcher with expertise in FHT and qualitative research" |
| 3. | Occupation | What was their occupation at the time of the study? | Page 7, Line 174: "JMG is a female mid-career researcher and academic GP with experience in qualitative research. SC is an early-career researcher with expertise in FHT and qualitative research" |
| 4. | Gender | Was the researcher male or female? | Page 7, Line 174: "JMG is a female mid-career researcher and academic GP with experience in qualitative research. SC is an early-career researcher with expertise in FHT and qualitative research" |
| 5. | Experience and training | What experience or training did the researcher have? | Page 7, Line 174: "JMG is a female mid-career researcher and academic GP with experience in qualitative research. SC is an early-career researcher with expertise in FHT and qualitative research" |
| Relationship with participants | | | |
| 6. | Relationship established | Was a relationship established prior to study commencement? | Page 7, Line 175: "JMG did not have a previous relationship with any of the interviewees. SC had had previous contact with two practice staff from one practice as research coordinator for a previous study using FHT" |
| 7. | Participant knowledge | What did the participants know about the researcher? e.*g. personal* | Page 7, Line 177: "We did not provide information regarding personal goals or additional reasons for doing the research other than the description in the PLS." |

| | | | |
|---|---|---|---|
| | of the interviewer | *goals, reasons for doing the research* | |
| 8. | Interviewer characteristic s | What characteristics were reported about the interviewer/facilitator? e.g. *Bias, assumptions, reasons and interests in the research topic* | Page 7, Line 177: "We did not provide information regarding personal goals or additional reasons for doing the research other than the description in the PLS." |
| **Domain 2: study design** | | | |
| Theoretical framework | | | |
| 9. | Methodologi cal orientation and Theory | What methodological orientation was stated to underpin the study? *e.g. grounded theory, discourse analysis, ethnography, phenomenology, content analysis* | Page 8, Line 197: "Interview transcripts were uploaded into NVivo 14 (Lumivero, Denver, USA) and thematically analysed independently by two researchers (JMG and SC) [26]. We used deductive thematic analysis using two overarching frameworks in the analysis: Acceptability of Healthcare Interventions [27] and Sociotechnical Model for Evaluation of Digital Interventions Framework [22] (Figure 3)." |
| Participant selection | | | |
| 10. | Sampling | How were participants selected? *e.g. purposive, convenience, consecutive, snowball* | Page 6, Line 142: "Recruitment of general practices occurred by approaching practices participating in the FHT program (i.e., practices that already had FHT installed)."<br>Page 6, Line 148: "We invited practices that had at least five patients identified by the UWL algorithm via email" |
| 11. | Method of approach | How were participants approached? e.*g. face-to-face, telephone, mail, email* | Page 6, Line 148: "We invited practices that had at least five patients identified by the UWL algorithm via email" |
| 12. | Sample size | How many participants were in the study? | Page 9, Line 238: "We recruited five general practices from a pool of 40 practices that had the FHT Program installed." |

| | | | |
|---|---|---|---|
| 13. | Non-participation | How many people refused to participate or dropped out? Reasons? | No participants refused or dropped out, they just did not respond to emails. |
| Setting | | | |
| 14. | Setting of data collection | Where was the data collected? e.*g. home, clinic, workplace* | Page 10, Line 240: "We conducted five interviews with seven practice staff. Interviews were conducted via Zoom, after the list of patients had been reviewed by practice staff and at least a month had passed to give patients the opportunity to come back for a follow-up appointment if recalled by the practice". |
| 15. | Presence of non-participants | Was anyone else present besides the participants and researchers? | No one else was present |
| 16. | Description of sample | What are the important characteristics of the sample? *e.g. demographic data, date* | Page 10, Line 244: Table 2 |
| Data collection | | | |
| 17. | Interview guide | Were questions, prompts, guides provided by the authors? Was it pilot tested? | Yes. Scripts provided in the supplementary materials |
| 18. | Repeat interviews | Were repeat interviews carried out? If yes, how many? | Page 7, Line 179: "Participants were not asked to review interview transcripts or study findings, and no repeat interviews were conducted." |
| 19. | Audio/visual recording | Did the research use audio or visual recording to collect the data? | Page 7, 172: "All interviews were audio-recorded for analysis and transcribed using Otter.AI " |
| 20. | Field notes | Were field notes made during and/or after the interview or focus group? | Page 7. Line 180: "Field notes were not recorded during or after the interviews" |

| | | | |
|---|---|---|---|
| 21. | Duration | What was the duration of the interviews or focus group? | Page 7, Line 173: "Interview duration was 30-45 minutes." |
| 22. | Data saturation | Was data saturation discussed? | Data saturation does not apply in this case as we interviewed staff from all practices in the study. |
| 23. | Transcripts returned | Were transcripts returned to participants for comment and/or correction? | Page 7. Line 179: "Participants were not asked to review interview transcripts or study findings, and no repeat interviews were conducted" |
| **Domain 3: analysis and findings** | | | |
| Data analysis | | | |
| 24. | Number of data coders | How many data coders coded the data? | Page 8, Line 197: "Interview transcripts were uploaded into NVivo 14 (Lumivero, Denver, USA) and thematically analysed independently by two researchers (JMG and SC) [26]." |
| 25. | Description of the coding tree | Did authors provide a description of the coding tree? | Code tree not provided |
| 26. | Derivation of themes | Were themes identified in advance or derived from the data? | Page 8, Line 2198: "We used deductive thematic analysis using two overarching frameworks in the analysis: Acceptability of Healthcare Interventions [27] and Sociotechnical Model for Evaluation of Digital Interventions Framework [22] (Figure 3)." |
| 27. | Software | What software, if applicable, was used to manage the data? | Page 8, Line 197: "Interview transcripts were uploaded into NVivo 14 (Lumivero, Denver, USA) and thematically analysed independently by two researchers (JMG and SC) [26]." |
| 28. | Participant checking | Did participants provide feedback on the findings? | Page 7. Line 179: "Participants were not asked to review interview transcripts or study findings, and no repeat interviews were conducted" |
| Reporting | | | |
| 29. | Quotations presented | Were participant quotations presented to illustrate the themes / | In results: Pages 9-15. |

| | | | |
|---|---|---|---|
| | | findings? Was each quotation identified? e.*g. participant number* | |
| 30. | Data and findings consistent | Was there consistency between the data presented and the findings? | In results: Pages 9-15. |
| 31. | Clarity of major themes | Were major themes clearly presented in the findings? | In results: Pages 9-15. |
| 32. | Clarity of minor themes | Is there a description of diverse cases or discussion of minor themes? | In results: Pages 9-15. |

Tong A, Sainsbury P, Craig J. Consolidated criteria for reporting qualitative research (COREQ): a 32-item checklist for interviews and focus groups. International journal for quality in health care. 2007 Dec 1;19(6):349-57.
